# The Joyful Life: An Existential-Humanistic Approach to Positive Psychology in the Time of a Pandemic

**DOI:** 10.3389/fpsyg.2021.648600

**Published:** 2021-07-22

**Authors:** Brent Dean Robbins

**Affiliations:** Department of Psychology, Point Park University, Pittsburgh, PA, United States

**Keywords:** joy, emotion, happiness, gratitude, humility, eudaimonia, virtue

## Abstract

In the midst of a global pandemic, psychology has a duty to identify dispositional or character traits that can be cultivated in citizens in order to create resiliency in the face of profound losses, suffering and distress. Dispositional joy holds some promise as such a trait that could be especially important for well-being during the current pandemic and its consequences. The concept of the Joyful Life may operate as bridge between positive psychology and humanistic, existential, and spiritual views of the good life, by integrating hedonic, prudential, eudaimonic and chaironic visions of the good life. Previous phenomenological research on state joy suggests that momentary states of joy may have features that overlap with happiness but go beyond mere hedonic interests, and point to the experience of a life oriented toward virtue and a sense of the transcendent or the sacred. However, qualitative research on the Joyful Life, or dispositional joy, is sorely lacking. This study utilized a dialogical phenomenological analysis to conduct a group-based analysis of 17 volunteer students, who produced 51 autobiographical narrative descriptions of the joyful life. The dialogical analyses were assisted by integration of the Imagery in Movement Method, which incorporated expressive drawing and psychodrama as an aid to explicate implicit themes in the experiences of the participants. The analyses yielded ten invariant themes found across the autobiographical narrative descriptions: Being broken, being grounded, being centered, breaking open, being uplifted, being supertemporal, being open to the mystery, being grateful, opening up and out, and being together. The descriptions of a Joyful Life were consistent with a meaning orientation to happiness, due to their emphasis on the cultivation of virtue in the service of a higher calling, the realization of which was felt to be a gift or blessing. The discussion examines implications for future research, including the current relevance of a joyful disposition during a global pandemic. Due to the joyful disposition’s tendency to transform suffering and tragedy into meaning, and its theme of an orientation to prosocial motivations, the Joyful Life may occupy a central place in the study of resiliency and personal growth in response to personal and collective trauma such as COVID-19.

## Introduction

In the midst of a global pandemic due to COVID-19, some might view the study of the joyful life as a frivolous investigation and perhaps even as a waste of resources. One might say that psychological investigators would better spend time on more pressing social and psychological problems of our day. On the contrary, however, a study of the joyful life, as understood within the humanistic and existential traditions, may be more relevant today than at times when things seem to be going right. When things go as planned, our senses can be respectively dulled by a sense of safety, security and normality. It is perhaps in times of trial and tribulation that one’s character is most tested for a capacity for an enduring and sustainable joyful disposition (Wong, 2011). From an existential perspective, for example in the philosophy of Martin Heidegger (1962), moments that emerge within a mood of anxiety or angst tend to reveal the world as uncanny. As a result, existence which is normally taken for granted can be recognized through a powerful sense of awe and wonder, opening the way to what Heidegger referred to as “unshakeable joy” (Robbins, 2012).

The study of joy, as a positive state or disposition, can be situated within the positive psychology movement (Emmons, 2020). As with the study of other pleasant emotional states and dispositions, the study of joy can be investigated under the “three pillars” of positive psychology identified by Seligman and Csikszentmihalyi (2000): the study of positive states, traits and institutions. Joy can be understood as a positive state or trait, and it can be studied for its implications for the cultivation of positive organizations, communities and societies (Seligman and Csikszentmihalyi, 2000).

Johnson (2020) suggested joy can be a subject of inquiry at four different levels of analysis: as an emotion, a mood, a trait or a spiritual fruit. Whereas the emotion of joy seems to operate thematically around issues of agency and perception of good or positive circumstances, joyful moods transcend particular circumstances. A joyful disposition or trait highlights a person’s tendency to be orientated to a sense of joy more often than is typical. When understood as a spiritual fruit, Johnson (2020) observed, the spiritual frameworks for understanding this type of joy express its aspect of transcendence, timelessness, and/or resiliency despite one’s circumstances. Even during times of suffering, crisis or challenge, the spiritual fruit of joy remains persistent in the agent’s perception of a potential for hope, meaning or transcendence within any given moment of challenge or difficulty. Therefore, when understood as a trait or spiritual fruit, a joyful disposition seems to be a character trait that holds promise for building resiliency in times of distress, such as the COVID-19 global pandemic. A joyful disposition may be found to be a trait that encourages post-traumatic growth – enhancement of self and relationships, and spiritual and existential growth during and after traumatic events (Cann et al., 2010).

### Building Bridges Between Positive Psychology and Humanistic, Existential and Spiritual Traditions

Positive psychology has been criticized for its tendency to over-emphasize positive experiences, a “tyranny of positivity” (Held, 2004). In contrast, existential and spiritual traditions have stressed a vision of the good life as a refusal to avoid negative experiences, since the suppression of life’s tragic dimensions, or “anesthetic consciousness,” can itself foster varieties of pathologies and dampen the vitality of life (Robbins, 2018, 2008). Alternatively, existential, humanistic and spiritual traditions tend to place emphasis on transforming the tragedy of life into meaning, hope for transformative liberatory practices, and virtue (Wong, 2011). Positive psychology seems most at risk of a superficial vision of the good life to the extent that it becomes reduced to a hedonic variety of happiness, which defines happiness in terms of a ratio of positive to negative experiences (Robbins, 2018, 2008), and de-emphasizes the moral or ethical dimensions of human flourishing in order to avoid explicit reflection on the value-laden project of the study of the good life (Friedman and Robbins, 2012; Robbins and Friedman, 2017; Yakusko and Blodgett, 2018; Prinzing, 2020).

As compared to hedonic concepts of happiness, such as satisfaction with life or the ratio of positive to negative affect, studies of joy, to date, suggest that joy can be meaningfully and empirically distinguished from hedonic happiness or momentary states of pleasure (Robbins, 2006, 2011, 2014). Matthewes (2015) has suggested that joy’s distinction from happiness lies in its tendency to emerge as a response to a solicitation from something larger than ourselves – into communion with others and a deeper, more engaged life (Emmons, 2020). The construct of a joyful life, therefore, may be a potential bridge between positive psychology and conceptions of the good life found in existential, humanistic, and spiritual perspectives on the good life.

### Visions of the Good Life

The richness and value-laden experience of a joyful life, with its spiritual/existential breadth and depth, offers to serve as a central conceptual foundation for what Wong (2011) has identified as “Positive Psychology 2.0.” Within this framework, conceptions of happiness have been cataloged into four types: hedonic, prudential, eudaimonic, and chaironic happiness (Wong, 2011). Hedonic happiness represents the much-criticized vision of the good life reduced to the ratio of pleasure to pain, and mere satisfaction with the circumstances of one’s life. Prudential happiness views flourishing through the model of active and agentic engagement in achievements, but lacks a moral or ethical dimension to its strivings. In contrast, eudaimonic happiness offers a vision of the good life founded on virtue and excellence, which relies upon dispositions and actions rightly ordered to the moral or ethical good. Chaironic happiness, finally, accords with the existential and spiritual traditions and their accent on a life founded on appreciation of one’s fortune, and a sense of awe or gratitude regarding the blessing or gift of life, in all its complexity and through all of its tragedies and triumphs.

Wong’s (2011) “Positive Psychology 2.0” recommends a view of human flourishing based in a “meaning orientation” which integrates eudaimonic and chaironic conceptions of happiness, for a more consummative perspective on the good life. In contrast to a “happiness orientation” which tends to be self-serving in its desire for hedonic and prudential well-being, and strives to avoid pain and challenges, the “meaning orientation” focuses instead on the actualization of meaning and purpose, the pursuit of rightly ordered goods even if this means personal sacrifice, and the striving for more enduring and valuable ends rather than simply momentary, fleeting pleasures.

### Spiritual, Existential and Humanistic Perspectives on Joy

Within the spiritual traditions of the West and East, joy has tended to be understood in accordance with a “meaning orientation” to the good life (Robbins, 2014). Within the Judeo-Christian tradition, for example, joy is typically described as a transformative experience in which suffering and despair are substituted by celebration and gratitude through the grace of God (Moltman, 2015). For example, in the Letter to the Hebrews, Paul foretold that the discipline of virtue necessitates pain and suffering, but their agony would be transformed into joy by the “peaceful fruit of righteousness to those who are trained in it” (Hebrews 12:11). Enduring joy, therefore, will not be found by attachment to fleeting pleasures, but rather “only as a feature of a flourishing life, a life lived well and that goes well…” (Moltman, 2015, pp. 10-11). Similarly, within the Islamic tradition, hedonic happiness, which is momentary and superficial, is identified by the Arabic term, *farah*, the brief “enjoyment” of those who “rejoice in the life of this world” (Al-Ra’ad, 13:26). However, the enduring state of joy is designated in Arabic as *‘sa’adah*, which can be found only in the presence of God (Ghazi bin Muhammad, 2014). In the Eastern tradition of Buddhism, as outlined in the *Mettanisama Sutta* (Klein, 2014), joy is designated as *mudita*, one of the four boundless qualities or four Brahma dwellings which lead to enlightenment. The other qualities of equanimity (*upekkha*), love (*metta*), and compassion (*karuna*), serve to liberate the ego from its limits. Joy or *mudita*, in particular, means to rejoice in all beings who are freed from suffering, a state of mind necessary for the culmination of the final Buddha wisdom, which transforms intention into action (Klein, 2014). While Judeo-Christian, Islamic and Buddhist traditions certainly have their differences, they nevertheless share an appreciation for joy as a necessary condition for spiritual well-being and fulfillment. More importantly, their vision of the good understands joy as a condition brought about primarily through the pains and tragedies of life, which are transformed into virtue through struggle, suffering and ultimately Divine grace or spiritual liberation. The pervasive theme of joy across major world religions, whether theistic or non-theistic, or Western or Eastern in origin, suggests that a distinction between momentary hedonism and a joyful disposition is grounded in the existential condition of human beings, and therefore not merely historically or culturally contingent concepts.

In accordance with the spiritual traditions outlined above, existential and humanistic approaches also make a similar distinction between fleeting hedonic pleasure and an enduring joyful disposition (Robbins, 2011, 2014). Importantly, the existential and humanistic traditions offer a view of the joyful life that is broader than theistic and overtly religious viewpoints, and therefore offer a potential vision of the good life available to individuals who are not committed to any particular religious or spiritual viewpoint. Rollo May (1981), for example, understood joy as the delight in sheer possibility, even in the face of despair, in contrast to mere pleasure-seeking in any given moment. Abraham Maslow’s later work, clarified that the height of human flourishing, which he identified as self-actualization, was better characterized as self-transcendence (Koltko-Rivera, 2006). By self-transcendence, Maslow was referring to the importance that a joyful and meaningful life was only actualized to the extent that any given person had become less focused on the self and more focused on serving others or a greater good beyond the self. Maslow’s vision of the good life had benefited from the influence of his colleague, Frankl (1966), who likewise stressed a view of the meaningful life as one not disposed to equilibrium or homeostasis as much as toward the productive tension of a “will to meaning” that was fundamentally oriented beyond the self – in the service of a meaningful project, the love of others, and/or toward the ultimate meaning of a transcendent spiritual dimension. Inspired by Frankl, Wong (2016) has also stressed the self-transcendent quality of the “meaning orientation,” which cultivates meaning through mindful attention in the moment, the pursuit of a meaningful calling in life, and the quest for ultimate meaning within the spiritual life.

### Empirical Research on State Joy

Approaches to the study of state joy tend to take either a dimensional approach or conceptualize emotions as discrete entities, although some investigators suggest dimensional and discrete approaches can be complimentary (Harmon-Jones et al., 2017). Dimensional approaches emphasize elements of emotional states, such as arousal, valence, and motivational direction. Discrete emotion approaches operate from the assumption that certain emotions are basic, in the sense of being rooted in early development, shared to some extent with other organisms in our evolutionary history, and correlated with distinct biological signatures (Harmon-Jones et al., 2017).

Arousal is one dimension that has been used to explore the nature of joyful emotional states. The dimension of arousal may be operationalized as the degree of physiological arousal within the autonomic nervous system or as the subjective experience of being in a state of arousal. Correlations between physiological arousal and subjective experiences of arousal may vary (i.e., Grueppel-Klein, 2005). For example, individuals in depressive states have been found to rate their physiological arousal to be more subjectively intense than a non-depressed control group (Wenzer et al., 2017). Whereas some investigators have operationalized joy as a state of high arousal (Neuman and Waldstein, 2001; Kuppers, 2008), there is some evidence that sub-forms of joy may include an excited joy type and a relaxed joy type (Wolf et al., 2005), though the latter has been categorized as “serenity” in distinction from “joy” (Clark et al., 1984). Facial expressions of joy have been found to differ from other emotions as a function of the activation of the M.orbicularis and M.zygomaticus muscles. Excited joy as opposed to relaxed joy or “serenity” was distinctive due to activation of the M.depressor anguli oris, which was linked to increased arousal as well as a joyful, positive valence (Wolf et al., 2005). Joy has been operationalized as an emotional state characterized by both a positive valence and high arousal (Russell, 1980; Larsen and Diener, 1992; Neuman and Waldstein, 2001). Emotion valence is best defined as the subjective feel of the emotional state, whether positive or negative, pleasant or unpleasant to the person (Ekman, 2003; Harmon-Jones E. et al., 2011; Harmon-Jones and Gable, 2018). The association of positive emotion with positive valence and high arousal has been found to have linguistic correlates across variations in languages, genders, handedness, and ages. Namely, positive emotional states tend to be expressed using metaphors that locate them within an upward spatial direction, whereas negative emotions tend to be expressed in terms of downward spatial locations (Marmolego-Ramos et al., 2017). However, positive valence alone does not appear to distinguish joy from other positive emotional states, including amusement, awe, interest, pride, love, gratitude and contentment, which seem to share positive valence as a dimension (Campus et al., 2013). Nevertheless, one study found electromyography was able to distinguish joy from schadenfreude (pleasure in the suffering of another), due to relatively stronger reactions (arousal) in schadenfreude as compared to joy, and higher pleasure (valence) in the joy condition relative to schadenfreude (Boecker et al., 2015). Joy’s positive valence (and the pleasant quality of other positive emotions) accounts for neuroscientific evidence correlating joyful states with activation of brain regions linked to hedonic and appetitive functions (Burgdorf and Panksepp, 2006; Takahashi et al., 2008; Colibazzi et al., 2010; Kuhn and Gallinat, 2012; Koelsch and Skouras, 2014).

Evidence in affective neuroscience suggests that valence can be meaningfully distinguished from another dimension, motivational direction (Harmon-Jones and Gable, 2018). The motivational direction of an affect refers to its action tendency toward either approach or avoidance of a stimulus (Harmon-Jones et al., 2013). The distinction between valence and motivational direction is perhaps most apparent in the case of anger. Whereas anger shares a negative valence with other unpleasant emotions, anger’s motivational direction is approach-oriented rather than avoidant (Harmon-Jones and Gable, 2018). Neuroscientific evidence, replicated in various experimental studies with various novel techniques, demonstrate that approach-oriented emotions are associated with asymmetrical activation of left frontal cortical activity in the brain, whereas avoidant-orientated emotions, such as fear, contrarily involve asymmetrical right frontal cortical activation (Harmon-Jones and Gable, 2018). While many positive emotions are approach-oriented in their motivational direction, judgments of photographs by naïve judgments provide some evidence that joy is low in approach-motivation, in contrast to the state of determination (Harmon-Jones C. et al., 2011). Gable and Harmon-Jones (2008) suggest that joy is low in approach motivation since it is an affective state that arises in response to the achievement of a sought-after goal, whereas determination is high in approach motivation because it remains optimistically oriented to a goal that is yet to be achieved. These, and the above reviewed research, suggest that joy can be primarily distinguished as an affective state with positive valence, high or low arousal, and low motivation for approach or avoidance.

Other dimensional theories of emotion, with some evidence base, suggest that emotion might be characterized along three dimensions of pleasure/valence, arousal, and dominance (Russell and Mehrabian, 1977), along four axes that include pleasantness, sensitivity, aptitude and attention (Cambria et al., 2012), or by the three facets of joy, interest, and activation (Egloff et al., 2003). Russell and Mehrabian (1977) found that happiness was associated with extreme pleasure and to a lesser extent, also arousal and dominance. Cambria et al. (2012) identified joy with the dimension of pleasant valence. When joy or high pleasantness interacted with the dimension of attention, the emotional state ranged from frivolity (high pleasantness-low attention) to optimism (high pleasantness-high attention). When interacting with the dimension of aptitude, the model suggested joy would range from love (high pleasantness-high aptitude) to gloating (high pleasantness-low aptitude).

A number of researchers, using various methods, have identified joy as a basic or discrete emotion (Tomkins, 1962; Izard, 1977; Ekman, 1982; Plutchik, 1994). Tomkins’s (1962) theory of affect identifies enjoyment-joy as a basic emotion, along with five other pairs: interest-excitement, surprise-startle, distress-anguish, anger-rage, and fear-terror. He understood joy to be the more intense expression of enjoyment, with the affective pair orientated primarily to the maximizing of pleasure. Based on his studies of facial expressions of emotion, Ekman (1982) identified joy as the sole positive emotion that was recognizable across cultures. While joy has been consistently identified as a universal, basic emotion, rooted in early development, elicited by specific stimuli, and found in analogous expressions in non-human animals, a number of investigators have identified what they believe to be other discrete positive emotions, including interest or anticipation (Tomkins, 1962; Izard, 1977; Plutchik, 1994) and acceptance-trust (Plutchik, 1994). In the research of De Rivera et al. (1989), they identified evidence that joy may be meaningfully distinguished from elation and gladness based on their eliciting conditions. Whereas joy was linked to a meaningful encounter with a unique other, elation and gladness were associated with the achievement of a wish or hope, respectively. In a study of college students’ pleasurable emotions in response to various conditions, a factor analysis suggested joyful or pleasurable emotions could be categorized into three types: cheerfulness, contentment, and enchantment (Berenbaum, 2002). Also, in an experimental examination of appraisal patterns among positive emotions, Ellsworth and Smith (1988) found that appraisals of effort, agency, and certainty differentiated joy from interest, hope-confidence, challenge, tranquility, and playfulness. The wide variations in nosologies of basic positive emotions implicate the need for further inquiry, although recognition of joy as a basic emotion seems to be a virtual consensus among investigators of affect.

Developmental research provides additional evidence that joy is a basic emotion, because it emerges early in infancy (Aksan and Kochanska, 2004). While observing infants interact with various toys and during a game of peek-a-boo with adults, factor analysis of videotaped observations identified joy as an expression that emerged in both social and non-social conditions (Aksan and Kochanska, 2004). Joy, therefore, seems to function at times as a means to coordinate pleasant social interactions, while at other times, reinforcing the infant in response to playful mastery of the external world. Similarly, one-year-old chimpanzees have been observed exhibiting emotional expressions of joy (play face and bodily expressions) in response to both social and solitary play (Ross et al., 2014). Collectively, this evidence suggests that joy occurs early in human and primate development, likely functions as a reward for social bonding as well as achievement, and corresponds to playful behavior.

To the extent that joy is a basic emotion, one could argue that, at least early in development, it operates as a “natural kind” – that is, “a category of phenomena that are given by nature, having similar observable properties, and are alike in some significant way” (Izard, 2007, p. 261). Like other basic emotions, joy can be designated as a “basic emotion” and “natural kind” in that it emerges early in development, evolved in the form of recognizable bodily expressions and internal neural mechanisms, is predictably elicited by certain environmental stimuli, has a distinct felt sense linked to neurobiological processes, functions to regulate cognition and action, and serves to motivate adaptive behavior (Izard, 2007). However, as the individual matures, basic emotions such as joy develop in interaction with higher-level cognitive functions, by which the basic emotion is transformed into more complex and variable emotion schemas. In the mature person, therefore, joy and similar basic emotions may no longer qualify as natural kinds, due to their variability as a function of the interpenetration of the basic emotion with cognition, such as the capacity to form concepts and complex perceptions. When occurring with a certain frequency, joy may co-occur in a stable manner with emotion schemas to such an extent that it begins to operate as an emotion trait, or as the motivational foundation for a person’s distinct personality. In such cases, joy may become a stable personal trait—a joyful disposition—rather than only a discrete, temporary emotional state (Izard, 2007).

### Phenomenological Research on Joy

Phenomenological, qualitative research on the state of joy provides some evidence in support of the claim that a joyful state has, in essence, a “meaning orientation” (Robbins, 2006). Chaironic qualities of state joy included a sense of awe or wonder, as well as a sense of gratitude for one’s existence (Robbins, 2006). This finding has been validated in quantitative studies demonstrating a correlation between state and trait gratitude and state and trait joy (Watkins et al., 2018). Participants higher in dispositional gratitude tended to have more frequent states of joy, and correlatively, the frequency of state gratitude was linked to dispositional joy. In addition, trait joy was found to predict increases in subjective well-being over time, as well as greater spiritual well-being. Also in keeping with the assumptions of phenomenological inquiry on joy as a distinct phenomenon, Watkins et al. (2018) found that joy operated as a distinct positive emotion.

Another phenomenological theme in experiences of trait joy was a sense of being balanced or centered in one’s body or lived world (Robbins, 2006). This finding dovetails with Meadows’ (2014) empirical finding that a sense of harmony or unity was a hallmark phenomenological dimension of joy. Also in keeping with Meadows’ (2014) observation that joy was correlated with a sense of energy, potency and aliveness, as well as a pleasant and rewarding experience, qualitative findings identified a warm, outwardly directed experience of energy or motivation, that was described as a movement up and out of the experiential body toward the world (Robbins, 2006). The warmth and energetic dynamic of joy was experienced as culminating in a felt connection to others, the world or a transcendent spiritual dimension beyond the self. Similarly, Meadows (2014) found that a sense of transcendence was among the phenomenological dimensions of joy. Transcendence in this sense was described, on the one hand, as a shift out of ordinary experience or consciousness, and, on the other hand, as a transcendence of ego-consciousness, ordinary time and space, and/or the past. This latter finding also accords with Robbins’ (2016) observation that participants described joy as a transformation of the perception of space and time, in which the participants experienced something like a sense of eternity or complete immersion in the present moment, so that the usual flow of temporal experience was felt to be expansive and uncontained by the usual linear structure of the clock. Participants also described a paradoxical sense of freedom as well as a loss of control or agency, or alternatively, the dropping away of the means-end goal orientation of typical ego-consciousness directed toward extrinsic desires (Robbins, 2006; Meadows, 2014). Finally, investigations by Robbins (2006) and Meadows (2014) converged on the finding that joy was characterized by shifts in perception, including more vivid sensations, a broader perceptual field of vision, and expansive motor movements.

The phenomenological dimensions of joy, originally identified by Robbins (2006) but independently validated by Meadows (2014), point to various themes associated with Wong’s (2011) “meaning orientation” to the good life. The chaironic themes of spiritual transcendence, especially with the emphasis on awe, wonder and appreciation for life, were salient in both investigations. In addition, the felt movement of state joy seemed to orient the participants to a self-transcendent state, both in the sense of a transformation of ordinary consciousness and a directedness beyond the self toward important others, projects and/or spiritual dimensions of reality. One could say the state of joy represented a break from the usual, bounded ego-orientation of instrumental engagement, which could perhaps be characterized almost as a forgetting of the self due to a hyper-focus on a world beyond or transcendent of the self. Watkins (2020), along these lines, has suggested that joy’s distinct appraisal process is distinguished by a sense of closer connection to another person or project deemed important, valuable or good. The orientation toward a valued transcendent “other” implies a moral or ethical dimension intrinsic to joy, in keeping with a more eudaimonic aspect of well-being. However, due to the fact that this research has focused on state rather than trait joy, it would be a stretch to suggest such momentary states indicate any enduring fulfillment or actualization of virtue.

### The Need for Qualitative Research on Dispositional Joy

Inquiry into the relevance of joy for a “meaning orientation” to the good life necessitates looking beyond transitory states of joyful emotion, and requires investigation into dispositional joy. If the joyful life is a qualitative expression of a “meaning orientation” to the good life, in keeping with spiritual, existential and humanistic traditions, this would need to be explored and identified in descriptions of dispositional joy – within the lived experience of participants in their description of the good life understood within the chairotic and eudaimonic sense of fulfillment. Therefore, an ideal place to start such an investigation, and the focus of this study, will be a phenomenological analysis of the experience of dispositional joy. The inductive nature of the qualitative inquiry of an empirical phenomenological investigation affords science with an opportunity to allow the description of the lived experiences of participants to form the basis for the identification of common thematic dimensions of the joyful life. Once a phenomenological investigation has been conducted, the general thematic findings can serve as the basis for the development of a scaling instrument to measure the joyful life construct based on the inductive analysis of narrative descriptions of participants, grounded in their lived experience.

It is reasonable and would be validating for qualitative findings of the joyful life to find corroboration and support via overlapping themes found in research on various other psychological constructs of mental well-being and human flourishing. While an exhaustive review of research on mental well-being is beyond the scope of this literature review, several constructs are worth mentioning, to the extent that they share a conception of the good life that integrates eudaimonic and chaironic elements.

### Subjective Well-Being

Subjective Well-Being (SWB) is the most commonly studied psychological construct in the study of happiness and the good life (Pavot and Diener, 2008; Diener et al., 2018). SWB is conceptualized as possessing a three-component structure including positive affect, negative affect, and life satisfaction (Andrews and Withey, 1976; Diener, 1984; Arthaud-Day et al., 2005). The best measures of SWB tend to combine judgment-based measurements, such as satisfaction with life, with measurements of affect (Diener et al., 2018). Satisfaction with life has been defined as a “cognitive and global evaluation of the quality of one’s life as a whole” (Pavot and Diener, 2008, p. 137). SWM has been found to correlate with relationship and marital satisfaction, material well-being, religiousness, and health; although, these relationships are highly contextual and mediated (Diener et al., 2018). SWB has also been consistently linked to personality traits; for example, neuroticism was found to be the most robust predictor for life satisfaction, and extraversion and agreeableness were most strongly linked to positive affectivity (DeNeve and Cooper, 1998).

Due to its focus on pursuit of positive affect, avoidance of negative affect, and cognitive evaluations of life satisfaction, SWB had been characterized as a hedonic conception of well-being (Ryan and Deci, 2001). On the one hand, it seems reasonable to predict that descriptions of a joyful life, or a joyful disposition, would include frequent and intense experiences of positive affect, and therefore would have a hedonic component. On the other hand, as already noted above, spiritual and existential perspectives have made a conceptual distinction between a good life conceptualized as pleasure-oriented and one that is marked as joyful. A life oriented primarily to pursuit of pleasure, without regard for pursuit of intrinsic values nor cultivation of excellence, has traditionally been understood to be an impoverished and superficial vision of the good life. Consequently, descriptions of the joyful life may, alternatively, include not only a hedonic component, but also themes related to human flourishing, realization of intrinsic values, pursuit of moral goodness, triumph in the development and application of strengths and virtues, and a capacity to grow from tragic circumstances rather than tendencies to avoid suffering.

In keeping with the vision of Wong’s Positive Psychology 2.0, a joyful life may reveal a life that embraces unavoidable suffering, and a capacity to transform tragedy into creative avenues for personal transformation and growth (Wong and Bowers, 2018). If so, the capacity to face suffering, as a hallmark of a joyful life, may constitute a more stable vision of the good life based on a joyful disposition lacking in defensive or neurotic avoidance of life’s inevitable challenges and pain. This capacity to maintain approach-related coping in the face of challenges and threats is protective of positive affect, whereas avoidance strivings tend to be debilitating for hedonic well-being (Coats et al., 1996; Elliot et al., 1997, 2011). Indeed, life satisfaction tends to be buffeted by bottom-up influences (Heller et al., 2004), and at least in some cases, can be rather unstable (Fujita and Diener, 2005), perhaps due to individual differences related to defensive or avoidance goal-striving and/or experiential avoidance (Kashdan and Breen, 2007; Van Dijk et al., 2012). A joyful disposition may be protective of life satisfaction and positive affectivity, perhaps by buffering the negative impact of life events through approach-oriented coping rather than avoidant or defensive coping. This may be accomplished, ironically, by a willingness to face suffering and painful emotion rather than seeking to escape it.

### Psychological Well-Being, or Eudaimonic Well-Being

The concept of “eudaimonic” well-being, as noted above, has been contrasted with hedonic well-being (Heintzelman, 2018). Inspired by the ethics of Aristotle (2001) in his *Nicomachean Ethics*, eudaimonia has been defined “a reflection of virtue, excellence, and development of one’s full potential” and “refers to that which is worth pursuing in life” (Heintzelman, 2018, p. 2). Evidence suggests eudaimonia is not only conceptually but also empirically distinct from hedonic well-being (Keyes et al., 2002). A weakness of the application of eudaimonia in contemporary psychology is a lack of agreement on its definition (Kashdan et al., 2008). As Martela and Sheldon (2019) reported, the construct of eudaimonia has been operationalized “in at least 45 different ways, using measures of at least 63 different constructs” (p. 458). Nevertheless, some of the more well-researched measures of eudaimonia may prove to have significant thematic overlap with the construct of the joyful life.

One of the more frequently cited constructs related to eudaimonia is Carol Ryff’s psychological well-being (Ryff, 1989, 2018). Similar to the concept of the joyful life, Ryff’s measure of psychological well-being was informed by humanistic and existential theories of human flourishing, as well as from insights derived from clinical, developmental and social psychology (Ryff, 2018). Ryff’s (1989) seminal work conceptualized and operationalized psychological well-being along six dimensions: self-acceptance, positive relations with others, autonomy, environmental mastery, purpose in life, and personal growth. The latter two dimensions, purpose in life and personal growth, have been found to decline between mid-life and old age, and evidence suggests psychological well-being is related to the quality of one’s work and family life (Ryff, 2018). Purpose of life has been shown to be predictive of longevity (e.g., Cohen et al., 2016) and is protective against health risks in late life (e.g., Kim et al., 2013). Psychological being was found to reduce cortisol, which facilitates the functioning of reward circuitry in response to positive stimuli (Heller et al., 2013).

Eudamonia has also been defined in terms of self-realization and personal expressiveness (Waterman, 1993), social well-being (Keyes, 2002), motivation toward self-improvement (Huta, 2015), psychosocial integration, ego development, personal growth (Bauer et al., 2008), flow experiences, meaning-making (Delle Fave et al., 2011), and the outcomes of personal strengths and virtues (Seligman, 2002) (see Heintzelman, 2018, for a review of these concepts of eudaimonia). The Questionnaire for Eudaimonic Well-Being identifies six dimensions of flourishing, including self-discovery, perceived development of one’s best potentials, sense of purpose and meaning in life, investment of effort in pursuit of excellence, intense involvement in activities, and enjoyment of personally expressive activities (Waterman et al., 2010). The Comprehensive Inventory of Thriving uses six dimensions of eudaimonia, including subjective well-being, support and enriching relationships, interest and engagement in activities, meaning and purpose in life, mastery and accomplishment, control and autonomy, and optimism (Su et al., 2014). A few other measures of eudaimonia include, for example, the Mental Health Continuum (Keyes, 2002), the Personally Expressive Activities Questionnaire (Waterman, 1993), and the Flourishing Scale (Diener et al., 2010).

A review of the empirical literature suggests that hedonic and eudaimonic well-being are closely related, but, based on factor analyses, hedonic and eudaimonic well-being tend to load on separate factors (Heintzelman, 2018). Examination of the shared variance between hedonic and audaimonic well-being indicate this shared variance constitutes a general factor, an operational definition that may be close to what participants understand in terms of living a joyful life (Chen et al., 2013). Generally, eudaimonic pursuits seem to be more motivated by meaning whereas hedonic activities appear to reflect pursuit of pleasant affective states (Waterman, 1993; Waterman et al., 2008; Steger et al., 2008; Huta and Ryan, 2010). With that said, positive affective states may operate to prime judgments of meaning in life (King et al., 2006). In addition, purely hedonic motivation seems to lead to short-term benefits, whereas eudaimonic pursuits tend to yield more long-term benefits (Huta and Ryan, 2010). Empirical evidence indicates that roughly half of participants report hedonic well-being, whereas flourishing, defined as a combination of hedonic and eudaimonic well-being, is more rare, representing about 18% of participants (Keyes and Annas, 2009).

The joyful life may reflect thematic overlap with both hedonic and eudaimonic well-being, which is suggested by research on folk conceptions of happiness that tend to understand the moral quality of a person’s life as integral and even essential to happiness (Phillips et al., 2017). It may also be the case, as suggested above, that descriptions of the joyful life may reflect not only hedonic and eudaimonic themes, but also themes of chaironic well-being, including perceptions that life is a gift, a belief that one is blessed or fortunate, and feelings such as wonder, awe and gratitude in response to this realization (Wong, 2011).

### Positive Orientation

The construct of positive orientation has been conceptualized as a general factor accounting for the shared variance of pleasure and meaning striving, or hedonic and eudaimonic well-being, respectively (Oles and Jankowski, 2018). Positive orientation is defined as a combination of beliefs about the self, life, and the future, which are represented by the dimensions of self-esteem, satisfaction with life, and optimism (Caprara et al., 2010). Empirical evidence has supported the beliefs-affect-engagement model (Laguna, 2019). According to this model, active engagement and activity persistence is motivated by positive affect, whereas positive affect is primarily influenced by positive beliefs. The structure of positive orientation, as a common factor integrating self-esteem, life satisfaction, and trait optimism, has been shown to be cross-culturally valid based on data from Japan, Germany, and Italy, and has been found to predict benefits for health, well-being, and achievement (Caprara et al., 2012). The enhancement of functioning associated with positive orientation appears to be a consequence of the facilitation of positive affective states, active engagement, and self-efficacy (Caprara et al., 2019). To the extent that positive orientation and a joyful disposition overlap, incorporating both hedonic and eudaimonic dimensions into a general factor, qualitative descriptions of the joyful life will likely yield similar themes, i.e., vigorous and persistent activity, confidence in one’s ability to succeed, a hopeful and optimistic outlook, etc.

### Psychological Need Satisfaction and Self-Actualization

Abraham Maslow’s (1965) pioneering work on psychological well-being identified self-actualization as a need for “ongoing actualization of potentials, capacities, talents, as fulfillment of mission (or call, fate, destiny, or vocation), as a fuller knowledge of, and acceptance of, the person’s own intrinsic nature, as an increasing trend toward unity, integration, and synergy within the person” (p. 25). Maslow’s theory hypothesized that fulfillment of basic deficit needs for physiological nurturance, safety, love and belonging, and self-esteem would enable self-actualization through an emergent motivation for “being needs” representative of a fully functioning person (Maslow, 1993).

Maslow’s (1970) research on self-actualized individuals identified 15 characteristics common among highly functioning people, including: efficient perception of reality, acceptance of self and others, spontaneity, problem centeredness, detachment, autonomy, continued freshness of appreciation, mystical experiences or other oceanic feelings, human kinship, deep and profound interpersonal relationships, humility and respect, discrimination between means and ends, creativeness, resistance to enculturation, sense of humor, values, and ethics (as adapted from Cofer and Appley, 1964, pp. 669-670, and Maslow, 1970, pp. 128-149, a cited by Fernando and Chowdhury, 2015, p. 6). Qualitative descriptions of the joyful life, to the extent they overlap with descriptions of self-actualized individuals, should reveal similar, overlapping themes.

More recent empirical findings provide support for Maslow’s theory. Self-actualization has been found to be significantly and positively associated with mindfulness (Beitel et al., 2014), acceptance of self and tolerance of others (Parham and Helms, 1985), problem-focused coping (Hosseini Dowlatabad et al., 2014), increased autonomy (Bordages, 1989), trait gratitude (Emmons and Shelton, 2002), social support (Moore and Sermat, 1974; Ford and Procidano, 1990), secure attachment (Otway and Carnelley, 2013), marital satisfaction (Rowan et al., 1995), mental health (Ford and Procidano, 1990), felt originality (Yonge, 1975), endorsement of meta-values such as truth, goodness, perfection, justice and love (Mathes, 1978), conscientiousness (Brooker, 1976), lower material values (Kasser and Ahuvia, 2002), and civic engagement (Carver and Baird, 1998). Self-actualization has been found to predict a number of adaptive traits associated with psychological well-being, including enhanced self-esteem, more rational beliefs and behavior, higher extraversion, and lower neuroticism (Jones and Crandall, 1986). A more recent review of the literature by Kaufman (2018) found that self-actualization was correlated with a wide variety of constructs used to define the thriving individual, including life satisfaction, self-acceptance, positive relationships, mastery of the environment, personal growth, autonomy, purpose in life, and self-transcendence. Also, in a variety of domains, measures of self-actualization have predicted success in the achievement of both work-related and creative endeavors (Kaufman, 2018).

In the tradition of Maslow’s theory of need satisfaction, Martela and Sheldon (2019) have developed a model of psychological well-being based on psychological need satisfaction and informed by self-determination theory. Their model posits that the core of psychological well-being is the satisfaction of intrinsic needs, which mediates the relationship between eudaimonic strivings and subjective well-being. Drawing from a wealth of research on self-determination theory, Martela and Sheldon (2019) identify empirical support for intrinsic needs of autonomy (sense of environmental mastery), competence (the capacity to contribute to others) and relatedness with others (Ryan and Deci, 2017; Sheldon, 2018). These attributes remain ideal candidates for intrinsic needs essential to psychological well-being, for a variety of reasons. First, they contribute to positive affectivity. Second, they predict benefits to individuals over the long-term including improved health, personal growth, and adaptation to challenge. Third, these identified needs act as mediators between subjective well-being and eudaimonic strivings, as well as environmental conditions that promote or hinder adaptation. Finally, empirical support suggests these needs exist across various cultural contexts (Martela and Sheldon, 2019).

Moreover, the self-determination theory provides a parsimonious and well-established empirical foundation for a humanistic approach to psychological well-being, which remains consistent and supportive of the seminal work of humanistic theorists such as Maslow and Carl Rogers (Patterson and Joseph, 2007; Deci et al., 2013; DeRobertis and Bland, 2018). At the core of the theory is an appreciation for human motivation that is primarily oriented toward realization of its innate potential, grounded in an organismic wisdom, which can nevertheless be derailed or thwarted by the adoption of extrinsic motivations based on external pressures. As it is rooted in a humanistic and phenomenological approach, the life-world descriptions of the joyful life can be reasonably expected to dovetail with themes pervasive in these theories of human growth based on the satisfaction of psychological needs.

### Love of Life

Qualitative descriptions of the joyful life may also disclose thematic similarities to the “Love of life” construct, defined as “a general positive attitude toward one’s own life, a liking for it, and pleasurable attachment to it” (Abdel-Khalek, 2007, p. 125). The Love of Life Scale (LLS) includes items such as “Life deserves to be loved,” “Life seems beautiful and wonderful to me,” and “Life is a blessing whose value we should appreciate,” and includes elements of hedonic, eudaimonic, and chaironic happiness. The scale was found to have moderate correlations with measures of happiness and optimism, and weaker but positive and significant relationships with self-esteem and hope (Abdel-Khalek, 2007). Across various cultures, Love of Life has been found to be positively associated with happiness, satisfaction with life, physical health, mental health, and religiosity (Abdel-Khalek and Lester, 2010; Abdel-Khalek, 2011, 2012, 2013b, 2014; Abdel-Khalek and Singh, 2019), and was negatively related to depression and anxiety (Abdel-Khalek and Lester, 2010). Among a sample of men, Love of Life was predicted solely by trait extraversion, whereas women’s Love of Life scores were predicted by low psychoticism and neuroticism, as well as higher extraversion (Abdel-Khalek, 2013a). If the items on the LLS scale are predictive of qualitative descriptions of the joyful life, then this would suggest the emergence of perspectives on life as pleasurable, beautiful, wonderful, hopeful, satisfying, a treasure worth guarding, meaningful, and a blessing to be appreciated.

### Resilience and Hardiness

Resilience is a construct that refers to a capacity to manage environment stress and change, recover from adversity, and adapt in a successful way in order to achieve desireable outcomes despite encountering conditions that would typically frustrate achievement (Block and Block, 1980; Rutter, 1987; Garmezy, 1991; Masten, 2014; Di Fabrio and Kenny, 2015). Resilience may suggest either maintenance of personal stability through adversity, or even growth and flourishing in the face of hardships (Miller, 2003). Optimism, self-efficacy, and adaptability have been identified as protective resources that facilitate resiliency by fostering the individual’s sense of mastery, sense of relatedness, and regulation of emotional reactivity (Prince-Embury, 2007).

Resilience, like other constructs of psychological well-being, seems to hinge on the optimal functioning of affiliative systems of the brain and nervous system, which provide a capacity to navigate the person’s social environment (Feldman, 2020). Resources for resiliency such as plasticity, sociality and meaning may be strengthened in early development by healthy attachments that nurture capacities for empathy, perspective-taking, and intimacy (Feldman, 2020).

Resilience may operate as a higher-order virtue, similar to *phronesis* (wisdom), by coordinating personal strengths for adaptation to environmental and social challenges toward beneficent ends (Robbins and Friedman, 2011; Friedman and Robbins, 2012). To that extent, resilience may be considered an important, and perhaps even central constituent of eudaimonic well-being. Consequently, if the joyful life represents the life experiences of individuals who are especially virtuous and high in eudaimonic well-being, it would follow that themes of resiliency would emerge in qualitative findings.

Along these lines, evidence suggests that when individuals appraise stressful situations as challenging, higher resilient individuals are better able to make use of positive emotions to cope with stress (Kacmorek, 2009). Resilient individuals tend to use more proactive, task-oriented coping strategies than emotion-focused strategies, and this likely protects them from the maladaptive effects of emotion-oriented coping, which tends to increase negative affect and depression (Smith et al., 2016). Moreover, and in keeping with a joyful disposition framework, the relationship between adaptive coping with stress and resiliency has been found to be mediated by positive emotions (Vulpe and Dafinoiu, 2012), and positive emotions appear to build resiliency which, in turn, enhances satisfaction with life (Cohn et al., 2009). Similar findings have been observed in the case of the eudaimonic construct of psychological well-being, which is correlated with dispositional resilience (Sagone and De Caroli, 2014b). Resilient individuals tended to be higher not only in psychological well-being, but expressed their resiliency as a tendency to use problem-focused rather than emotion-focused or avoidant styles of coping (Sagone and De Caroli, 2014a).

An existential and humanistic framework for the study of resilience led to the development of the hardiness construct, which is conceptualized as a disposition toward “existential courage” (Maddi, 2004). Hardiness consists of three dimensions: commitment (“predisposition to be involved with people, things and contexts rather than be detached, isolated or alienated”), control (“struggles to have an influence on outcomes around oneself rather than sinking into passivity and powerlessness”), and challenge (“wanting to learn continually from one’s experiences, whether positive or negative, rather than playing it safe by avoiding uncertainties and potential threats”) (Maddi, 2002, p. 175). Like other constructs of resilience, hardiness is predictive of more adaptive and active forms of coping, social support, and enhanced performance, and negatively related to perceived stress and maladaptive, or avoidant forms of coping (Eschelman et al., 2010). Hardiness is positively related to hedonic well-being defined as satisfaction with life (Maddi et al., 2009; Civitci and Civitci, 2015) as well eudaimonic well-being operationalized as psychological well-being (Viola et al., 2016). Hardiness has also be found to be protective against depression, anxiety and hostility, likely by the existential courage with which to actively cope with stressful thoughts rather than avoidance of them (Maddi et al., 2009).

Given that the joyful life is grounded in an existential and humanistic framework, the theme of existential courage and the capacity to face and maintain engagement with challenges of life, in order to learn and grow from them, may be salient in the qualitative data.

### Mindfulness

Mindfulness has been characterized as “non-judgmental awareness of and attention to moment-by-moment cognition, emotion, and sensation without fixation on thoughts of past and future” (Kiken et al., 2015, p. 41; in reference to Kabat-Zinn, 1990). As a state, mindfulness is cultivated through meditative and contemplative practices. As a disposition, trait mindfulness refers to a tendency to be mindful in an ongoing way in everyday life (Kiken et al., 2015). Mindfulness has been shown to be protective against stress, negative affectivity and mood dysregulation. Enhancing mindfulness through meditative practice has been shown to increase mindfulness and decrease distress (Kiken et al., 2015). Trait mindfulness can help to reduce cortisol response to stress in laboratory conditions, which reduced anxiety and negative affect (Brown et al., 2012). In an experimental condition where participants were exposed to death salience, mindfulness helped to decrease worldview defense and efforts to protect self-esteem, even though mindfulness was linked to increased attention to, and decreased avoidance of thoughts about death (Niemic et al., 2010). Mindfulness has been found to activate brain structures (anterior cingulate cortex, insular cortex, and prefrontal cortex) that aid self-regulation of emotion and to deactivate brain structures linked to distressing emotions (amygdala) (Wheeler et al., 2017). Also, long-term meditative practices can result in structural and functional changes in the brain that help to enhance attention, emotion regulation, and psychological well-being (Wheeler et al., 2017).

Empirical evidence supports the hypothesis that mindfulness functions to enhance both hedonic and eudaimonic well-being. Participants with dispositional mindfulness presented with increased positive affect, improved regulation of emotion, and self-acceptance, which in turn were protective against symptoms of depression (Jimenez et al., 2010). The capacity to savor the moment appears to interact with dispositional mindfulness to foster positive emotions and mental health (Kiken et al., 2017). Acceptance of present-moment experiences, as opposed to defensive avoidance, was found to be at the core of mindfulness’s capacity to increase positive emotions (Lindsay et al., 2018). Another important mediator in the relationship between mindfulness and hedonic well-being (life satisfaction) is the role of self-evaluation (Kong et al., 2014). Evidence supports a model whereby dispositional mindfulness produces increased self-esteem, which in turn improves satisfaction with life (Pepping et al., 2013). Even in brief experimental inductions of mindfulness, state self-esteem has been shown to increase as an effect of the mindfulness induction (Pepping et al., 2013).

Mindfulness has been found to be associated with a number of eudaimonic constructs as well, including hardiness (Yavuz and Dilmac, 2020), self-actualization (Beitel et al., 2014), autonomy (Parto and Besharat, 2011), self-esteem (Pepping et al., 2013), hope and optimism (Malinowski and Lim, 2015), emotional stability, conscientiousness (Giluk, 2009), cognitive empathy (Winning and Boag, 2015), psychological well-being (Brown and Ryan, 2003; Hanley A.W. et al., 2015), and satisfaction of basic psychological needs (Chang et al., 2015). A link between mindfulness and chaironic well-being is implied, for example, by correlates with spiritual well-being (Yavuz and Dilmac, 2020), daily spiritual experiences (Greeson et al., 2011), gratitude (Swickert et al., 2019), heartfulness toward self and others (Voci et al., 2019), and meaning and engagement with life (Garland et al., 2015).

In a study utilizing Peterson and Seligman’s (2004) Values in Action (VIA) classification of strengths and virtues, mindfulness was moderately to weakly correlated with most categories, including hope, bravery, curiosity, social intelligence, self-regulation, creativity, humor, love of learning, forgiveness, leadership, spirituality, perseverance, open-mindedness, appreciation of beauty, honesty, and kindness, and to a lesser extent, fairness, teamwork, and prudence (Pang and Ruch, 2019). One exception was modesty, which did not correlate with mindfulness nor any of its facets.

Given the consistent correlations between mindfulness and traits closely linked to hedonic, eudaimonic, and chaironic well-being, descriptions of the joyful life may reveal themes of mindful engagement, including absorption in the moment. In addition, as with the concept of the joyful life, the mindfulness construct has been closely identified with humanistic and existential theory and practices (Felder et al., 2014; Jooste et al., 2015; Felder and Robbins, 2016, 2021; Hanna et al., 2017; Hoffman et al., 2020).

### Spiritual Well-Being

The chaironic conception of well-being may be well captured by the construct of spiritual well-being. National Interfaith Coalition on Aging (1975) defined spiritual well-being as “the affirmation of life in a relationship with God, self, community, and environment that nurtures and celebrates wholeness” (p. 1, as cited by Ellison, 1983, p. 331). Spiritual well-being can be conceptualized as having two dimensions, one vertical, and referring to the quality of one’s relationship to God, and another that is horizontal, denoting a sense of life purpose and satisfaction linked to ultimate concerns that need not be overtly theistic (Ellison, 1983). The latter, horizontal dimension can be referred to as an existential dimension in reference to knowing “what to do and why, who (we) are, and where (we) belong” (Blaikie and Kelsen, 1979, p. 137, as cited by Ellison, 1983, p. 331). Consistent with this model of spiritual well-being, Ellison’s (1983) Spiritual Well-Being scale loaded along two factors, one being an overtly religious well-being and the other existential.

A more recent model of spiritual well-being, with an even broaden definition of the construct, was developed by Fisher (1998), and became the basis for the Spiritual Well-Being Questionnaire (SWBQ) (Gomez and Fisher, 2003). Enriched by an emerging body of literature on the construct, Gomez and Fisher (2003) defined spiritual well-being in the broadest terms, as:

…the affirmation of life in a relationship with oneself (personal), others (communal), nature (environment), and God (or transcendent other)…defined in terms of a state of being reflecting positive feelings, behaviors, and cognitions of relationships with oneself, others, the transcendent, and nature, that in turn provide the individual with a sense of identity, wholeness, satisfaction, joy, contentment, beauty, love, respect, positive attitudes, inner peace and harmony, and purpose and direction in life (p. 1976).

The scale was designed and, as predicted, empirically validated Fisher’s (1998) model of spiritual well-being as operating along four domains, or factors: the personal, the communal, the environment, and the transcendental. The personal domain was composed of items indicating the development of a sense of identity, self-awareness, joy in life, inner peace, and meaning in life. Within the communal domain, the factor included items concerned with the development of love of other people, trust between individuals, respect for others, and kindness toward other people. Items within the environmental domain concerned growth in feeling a connection to nature, experiencing awe at a breathtaking view, a sense of oneness with nature, being in harmony with the environment, and having a sense of magic in the environment. Finally, the transcendental domain measured items regarding the development of a personal relationship with God, worship of the Creator, oneness with God, peace with God, and a prayer life (Gomez and Fisher, 2003). Global spiritual well-being demonstrated a weak but significant correlation with extraversion and happiness, and a moderate and significant negative relationship with psychoticism (Gomez and Fisher, 2003).

Subsequent investigations have found links between spiritual well-being and both hedonic and eudaimonic constructs. The positive effect of religiosity and spiritual on well-being appears to be mediated by self-transcendent positive emotions, such as awe, gratitude, love, and peace (Cappellen et al., 2016). In experimental manipulations inducing positive emotions with videos, religiousness and spirituality were given a boost by positive emotions, and in the case of spirituality, this effect was reserved only for self-transcendent emotions in response to videos of nature and childbirth (Saroglou et al., 2008). In experiments that induced elevation or admiration, self-transcendent emotions had the effect of increasing spirituality, most especially among participants who were not religious (Van Cappelen et al., 2013). The influence of positive emotions on spirituality was mediated, however, by worldview beliefs regarding the meaningfulness of life and the benevolence of others and the world (Van Cappelen et al., 2013). The relation between positive emotions and spirituality appears to be bidirectional, however, in that healthy spirituality has been found to predict positive emotions as well as resilience (Smith et al., 2012).

Spiritual well-being also correlates with eudaimonic psychological well-being, in addition to higher levels of happiness and reduced stress along the hedonic spectrum (Rowold, 2011). In a study of trait correlates with spiritual well-being, the construct was positively associated with sense of coherence, satisfaction with needs, extraversion, and openness, while negatively correlated with neuroticism (Unterrainer et al., 2010). Another study identified spiritual well-being as a predictor of perceived social support and life satisfaction (Van Direndonck, 2004).

### Posttraumatic Growth and Redemption Narratives

Posttraumatic growth references positive changes in the self, relationships, and one’s philosophy of life following a traumatic event (Tedeschi and Calhoun, 1996). Posttraumatic growth is associated with a number of indicators of well-being, including optimism, adaptive coping, social support, optimism, extraversion, openness to experience, agreeableness, conscientiousness, low neuroticism, emotional disclosure, optimism, and spirituality/religiosity (Ramos and Leal, 2013).

Positive affectivity seems to play an important role in posttraumatic growth (Chopko and Schwartz, 2009). Positive affect may mediate the relationship between posttraumatic growth and other indicators of flourishing, including perceived social support and resilience (Kong et al., 2018). Positive affects appear to combine with adaptive regulation of emotion and self-efficacy in the facilitation of posttraumatic growth (Yu et al., 2014. Also, reduced distress and posttraumatic growth were predicted by the eudiamonic variable of meaning in life as well as the hedonic indicator of life satisfaction (Triplett et al., 2012). In a sample of breast cancer patients, gratitude (a chaironic variable) predicted posttraumatic growth, psychological well-being, relaxation, and contentment, as well as reductions in anxiety, depression, and hostility-irritability (Ruini and Vescovelli, 2013). In the same study, participants with high gratitude, as compared to the low gratitude condition, were found to have greater posttraumatic growth and more positive affect (Ruini and Vescovelli, 2013). Posttraumatic growth has also been linked with the eudaimonic variable of relationship need satisfaction, as well as adaptive cognitions (i.e., challenge appraisals, acceptance, positive reframing) and emotional expressivity (Yeung et al., 2016).

Finally, posttraumatic growth seems to be facilitated by mindfulness, especially as expressed in relatedness to others, appreciation of life, changes in spirituality (Shiyko et al., 2017), regulation of behavior, and more adaptive evaluations (Hanley A.W. et al., 2015). Experienced mindfulness practitioners seem to benefit more greatly than inexperienced practitioners on outcomes of posttraumatic growth (Hanley A.W. et al., 2015).

Given that phenomenological methodology draws upon narrative descriptions of personal experiences, posttraumatic growth may appear as typical narrative structures in descriptions of the joyful life. Investigations of narrative identity support this presupposition (McAdams and McLean, 2013). Narrative identity is defined as a “person’s internalized and evolving life story, integrating the reconstructed past and imagined future to provide life with some degree of unity and purpose” (McAdams and McLean, 2013, p. 233). Within the narrative identity paradigm, posttraumatic growth has been shown to correlate with a typical narrative pattern, the redemptive narrative. The structure of the redemptive narrative begins with a tragedy or challenge in the life story of the narrator, but ends with a theme of triumph or positive transformation based on attributions of personal agency (Pals and McAdams, 2004; McAdams and McLean, 2013). Redemption narratives among midlife adults are more common among those with high generativity and psychological well-being, and these narrative structures seem to have a greater impact on well-being than even the affective qualities of the stories people tell (McAdams et al., 2001; Bauer et al., 2008). Joyful life narratives given in semi-structured interviews may likely include similar redemptive narrative structures.

### Extraversion and the Big Five

The Big Five traits have been associated with a variety of positive affective states, including joy, contentment, pride, love, compassion, amusement, and awe (Shiota et al., 2006). All of these positive emotions were positively associated with extraversion. These positive affective states, however, differed among the other Big Five traits. Conscientious was positively associated with joy, contentment, and pride; openness to experience was positively associated with joy, love, compassion, amusement, and awe; and neuroticism was negatively related to joy, contentment, pride, and love. Among these affective states, joy predicted the most variance across the four traits noted, and, with a correlation coefficient of 0.66, was the most closely tied to extraversion than any other affect. Lucas and Fujita (2000) identified a similar moderate to strong association between positive emotions and extraversion. These dispositions may be rooted in early attachment, since joyful emotion seems to be facilitated by secure attachment, lower levels of anxiety, and low preoccupied attachment styles (Shiota et al., 2006).

Extraversion is so closely linked to happiness that Frances (1999) suggested stable extraversion is the core of a happy disposition. The frequency, intensity and duration of positive emotions each predict extraversion, though duration appears to be the strongest predictor (Verduyn and Brans, 2012). Evidence suggests that state extraversion facilitates positive emotional states, even among trait introverts (Wilt et al., 2012). Extraversion may influence positive affect in two ways, through cultivating satisfying relations with others and by motivating environmental mastery, dominance and achievement (Watson and Clark, 1997). These pathways are remarkable in that they parallel the contexts of expressions of joy as a basic emotion in infancy. As noted above, expressions of joy among infants and chimpanzees tend to occur within the context of social interaction and mastery or achievement of tasks (Aksan and Kochanska, 2004; Ross et al., 2014). Consequently, narrative descriptions of the joyful life may also include frequent references to positive social interaction and achievement.

### Humility

As noted above, self-esteem has been incorporated into models of psychological well-being, and is linked to a variety of positive outcomes. However, self-esteem is notoriously difficult to tease apart from narcissism, even though the maladaptive consequences of narcissism are stark in comparison to the adaptive consequences of healthy self-esteem (Bosson et al., 2008; Tracy et al., 2009; Yonge et al., 2014; Brummelman et al., 2016). On the other hand, dispositional humility seems to be a distinguishing characteristic of healthy self-esteem which is absent in narcissism (Banker and Leary, 2020).

Indeed, humility seems to be protective in the face of stress, depression and anxiety, and is positively related to psychological well-being and life satisfaction (Krause et al., 2016). Humility may also be more strongly related to eudaimonic well-being than hedonic well-being (Aghababaei et al., 2016). Eudaimonic well-being has been demonstrated to be modestly related to both humility-cultivating practices and trait humility (Ruffing et al., 2021). The domains of openness and modest self-assessment appear to best account for its positive relationship to happiness (Sapmaz et al., 2016). Even when controlling for social desirability and trait differences, humility predicted high quality social relationships (Peters et al., 2011). Among religious leaders, humility helped to enhance mental health (Jankowski et al., 2019). Interestingly, results from a cross-lagged design suggest the direction of causality is from psychological well-being to humility rather than vice versa (Tong et al., 2019).

While humility has been operationally defined in various ways, one promising new avenue of investigation has explored humility as a form of “hypo-egoic non-entitlement” (Banker and Leary, 2020). In other words, at the core of trait humility is “the belief that one’s accomplishments and positive characteristics do not entitle one to be treated special as a person by other people” (p. 739). Humility as defined can, therefore, be contrasted with egoic entitlement, “the belief that other people should treat them differently as a person because of their accomplishments or positive characteristics” (p. 739). Bankey and Leary (2020) found that humility, or hypo-egoic non-entitlement, strongly predicted prosocial relations and identification with all of humanity. Humility, as defined, therefore, seems to represent a construct that perhaps overlaps domains of eudaimonic and chaironic well-being. If the joyful life incorporates these aspects of well-being, narrative descriptions should reflect humility rather than indications of egoic entitlement.

### Relevance to Life in a Time of a Pandemic

The study of dispositional joy can now be said to have importance and relevance in a contemporary world ravaged by a global pandemic and its social and economic consequences. Research on the mental health impact of COVID-19 has suggested that the mental health consequences of COVID-19 (as well as social policies aimed at containment) may outstrip the negative impact of the disease itself, if similar events in the past may be used as a guide to predict the future (Reardon, 2015). An investigation in China rated the psychological impact of COVID-19 as either severely or moderately impacting their mental health, with roughly a third of participants reporting moderate to severe anxiety (Wang and Zhao, 2020). Whether a patient is infected or suspected of having the virus, the emotional consequences can be quite devastating (Shigemura et al., 2020), and the distress can further develop into more severe, long-term psychopathologies, including depression, anxiety, psychosis, and even suicide (Xiang Y.T. et al., 2020). Patients in quarantine appear to be especially vulnerable to these psychological effects (Brooks et al., 2020). Even if individuals are not directly exposed to infection, the impact of uncertainty and worry about the future health and well-being of family members can take a toll on mental health (Maunder et al., 2003; Park and Park, 2020). Similar findings in Italy, the United Kingdom, and the United States demonstrated high rates of psychopathology compared to years of the recent past (Czeister et al., 2020; Pierce et al., 2020; Rossi et al., 2020).

Fortunately, even as the pandemic has been shown to drastically increase rates of distress and psychopathology in the general population, there is emerging data suggesting the existence of protective factors (Xiang J. et al., 2020). Among these protective factors are certain personality traits, including positive coping styles that suggest a resilient, joyful disposition may hold promise as a key to building resiliency to trauma, including future pandemics, in the general population. If dispositional joy can be shown to orient individuals to a sense of peace and well-being despite circumstances; if it moves them to engage in pro-social, self-transcendent action to help the common good; and if it provides a sense of meaning and orientation to a transcendent good beyond momentary pleasures, then: the good life defined as the joyful life may be a character state worthy of cultivating in a precarious and uncertain world during and beyond the current crisis of the global COVID-19 pandemic.

## Method

### A Dialogal Phenomenological Approach

Phenomenological approaches to qualitative inquiry offer a number of analytical strategies (Hein and Austin, 2001; Todres and Wheeler, 2001). Descriptive phenomenology draws upon Edmund Husserl’s transcendental phenomenology and its applications to psychology in order to identify general though situated thematic structures across narrative, first-person descriptions of participants (Giorgi, 2009). For Husserl, philosophical inquiries aim toward the discovery of universal eidetic insights into the essence of meaning, such as in the example of mathematics, which lend themselves to formal eidetic analysis. In the case of psychology and other social sciences, however, Husserl understood eidetic insights to discover not formal and universal structures, but “morphological essences.” While constitutive of meaning within a given historical, cultural or developmental context, “morphological essences” tend to vary through time and across varieties of experience in everyday life (Wertz, 2010). To uncover these stabilities of lived meaning, even as they are personally and culturally situated and in the process of ongoing transformation, requires a two-step process: First, a shift into a phenomenological attitude, and secondly, the eidetic reduction. The former step entails setting aside metaphysical assumptions about whether appearances exist in the mind or in the outside world, or traditional distinctions between subjectivity and objectivity, in order to describe the world (to the extent possible) just as it appears in perception (Robbins et al., 2018). The latter step of the eidetic reduction entails the use of imaginative variation of thematic constituents of experiences, in dialog with qualitative, experiential descriptions, which aims to disclose the general themes (“morphological essences”) that allow the phenomenon to disclose its lived structure (meaning) as it is experienced in everyday life (Giorgi, 2005).

This study utilized a dialogal phenomenological approach to inquiry, originally developed at Seattle University by Halling et al. (1994). This approach to qualitative inquiry involves the use of cooperative group dialog as a means to gain insight into general though situated thematic interpretations of the data. In this sense, the dialogal approach seeks out general situated and structural features of the phenomenon, while, consistent with the hermeneutic tradition, still recognizing these eidetic insights are always already embedded within the social, cultural and linguistic context of the group’s collective, interpretative understanding of the phenomenon (Macdonald, 2001; Laverty, 2003; Barua, 2007). As with Giorgi’s (2009) approach, the dialogal phenomenological method seeks out descriptions of eidetic insights at the psychological (rather than philosophical) level of analysis. Furthermore, in this study, analyses were embedded within the existential life-world of participants through a modification of the phenomenological method, which includes the interpretive framework that seeks variations in existential structures of human experience, including the lived experience of the body, others, time, space and place, language, and things (Robbins, 2006).

Because lived experience is pre-thematically and implicitly lived out prior to reflection and linguistic articulation, phenomenological inquiry is challenged to bring these experiences to explicit articulation through the use of analogies and other forms of creative speech that allow the implicit structure of experience to emerge into a structural description, including the outline of general themes and a wholistic description that attempts to capture the phenomenon as a whole (Wertz, 2016). This process requires the investigators to set aside or “bracket” their usual tendency, within the “natural attitude,” to discard experiences based on *a priori* presuppositions about whether experiences do or do not reference an objective reality. Instead, descriptions of the world are taken to be reflections of a lived world that is constituted as implicitly meaningful and motivated. As witnessed and described, first-person descriptions are indirectly reflective of structures of consciousness, including affective dispositions, which give rise to that perceptual (lived) world (Robbins, 2013). How people describe their world, in short, is an indirect reflection of the agent or self to whom that world is significant. As Giorgi (2002) put it, “Within phenomenology, the goal is not to try to eliminate subjectivity, but rather to try to clarify the role of subjectivity when correct knowledge is obtained” (p. 8). Or, as J.H. van den Berg (1974) expressed, “We get an impression of a person’s character, of his subjectivity, of his nature and his condition when we ask him to describe the objects which he calls his own; in other words, when we inquire about his world” (p. 13).

Group dialog facilitates the process of articulating lived experience by creating a forum in which participants can share their experience with others in the form of narrative descriptions. The subsequent interpretation of these narratives, in turn, emerges as a product of a group discussion in which differences and similarities among the group members challenge the participants to bring their experience into a clearer expression in order to communicate their experience to others (Halling et al., 1994).

Following the method outlined by Robbins (2006), which was modified for a dialogical phenomenological inquiry by a group of investigators, this study made use of the Imagery in Movement Method. Research-participants included 17 undergraduate student participants who engaged the phenomenological inquiry as part of the coursework in an undergraduate qualitative research course. The number of participants exceeds the recommended minimum number of participants for phenomenological research, which is 2-10 participants according to Boyd (2001) and Creswell (1998). The fact that the participants were restricted to university students is a limitation, although one that is shared by many of the seminal studies referenced in the literature review above. Use of students in dialogal phenomenology is typical and allows insight into phenomenological findings while also serving valuable pedagogical ends (Halling et al., 1994).

Participants included 15 female and 2 male investigators-participants, who were guided by the author (instructor) in the course of the analysis and interpretative dialogical process (The female-to-male ratio is noted to be proportionate to the female-to-male ratio of the program in which the students were enrolled). The author, however, took care, to the extent possible, to avoid introducing concepts or corrections to the participants’ descriptions, narratives, and interpretations. He strove to operate in the sole function of instructing participants in the process of description and analysis. Five of the participants identified as African-American, and the other 10 identified as white. The ages of the participants ranged from 20–24 years of age. All participants were psychology majors, and from within the region of Southwestern Pennsylvania. All were single and had never been married.

The homogeneity of the group, culturally speaking, operates both with its limitations and advantages. The limitations include the fact that generalization of the findings should be conducted with caution. An advantage, from within the context of a phenomenological study, is that the shared cultural horizons of the participants form a basis for some degree of mutual understanding and a capacity to make use of shared references. The primary aim for this study, therefore, is not necessarily to arrive at a universal and timeless understanding of the joyful life, but rather to explicate, in terms of cultural shared understandings, what would otherwise remain implicitly lived within the situated context of a culturally homogenous group. The extent to which the structural and thematic findings can be generalized beyond this cultural situation will require subsequent research.

In each case, the participants began their inquiry with spontaneous, abstract drawings of the joyful life with the use of Crayola markers and blank sheets of printing paper (5.5 × 11 inches in size). Participants were given the simple, open-ended prompt, “Please use color and form to express what it is like to live a joyful life.” This exercise has utility for several reasons. First, the use of abstract drawings encourages the participants to enter into a playful attitude conducive to the phenomenological attitude, including the adoption of verbal and visual analogies to express experiences for which they may otherwise have no readily available (formal or literal) vocabulary (Robbins, 2006). Also, the act of drawing on paper with markers tends to be an amusing exercise that is likely to induce a playful attitude and a joyful mood. Research suggests that memories of positive events and experiences are recalled more easily, directly and vividly if participants are in a positive emotional state (Sheldon and Donahue, 2017). Activities that induce positive emotions have been shown to aid recall of positive experiences through spreading activation of organization within the nervous system (Ford et al., 2012). Since the object of this research is to facilitate descriptions of vivid, autobiographical memories, the use of a task that is amusing and enjoyable promises to aid in the achievement of this end.

Together, as a group, the participants shared their artwork and assisted one another in a process of explicating thematic intrinsic themes in the drawing. Participants were instructed to start with an aspect of the drawing that was felt to be important and to verbalize how the colors and forms expressed their experience of living a joyful life. As the participants explored and shared their intuitions about the meaning of their drawing, particular memories of concrete experiences were evoked and described. The instructor encouraged the participants to elaborate upon these memories and express them verbally as autobiographical, narrative accounts.

To bring these experiences more vividly to the group, psychodrama techniques were used to role play the events that were being recalled and to make them more salient and visible to the group. The participants took turns standing at the front of the room, imaginatively described the scene of their autobiographical account, and enlisted volunteers among their peers to role-play parts in the story. These enactments of the story helped to make the experience come alive for the storyteller and the rest of the group. By watching the story unfold through these psychodramatic enactments, the group was better enabled to adopt an empathic, perspective-taking attitude through identification with the protagonist/storyteller and their experience. These psychodramatic enactments helped the participants to adopt a “reflexive embodied empathy” which facilitates perspective-taking by “tuning into another bodily way of being,” coupling with and mirroring the gestures of the other to get a felt sense of their point of view, and “merging-with” the experience of the other, as a potent source of insight for phenomenological inquiry (Finlay, 2005, p. 271).

Next, participants explored the metaphors and tropes that seemed to emerge in the drawings, the explication of the drawing’s themes, and in the re-living of recollected scenes brought back to life through role-play enaction. Participants used the markers and a new sheet of paper to list the dominant tropes that emerged in their accounts. After this process, the participant-investigators recorded their descriptions of memories into a narrative form, which was typewritten and shared with the instructor and group. These data, including the drawings, psychodramatic enactments, and narrative descriptions served as data for the reflexive, eidetical and hermeneutic analysis of the joyful life.

The process generated a total of 51 narrative descriptions of the joyful life. The excess number of descriptions were a product of the instruction to participants that they were permitted to produce as many drawings and narratives as they wished. While some participants chose to produce only one drawing and narrative, others opted to create several. Since findings in phenomenological research involve thematic regularities across the participants rather than an aggregate or mean quantitative score, the frequency of artifacts produced by the participants, in this case, simply provide additional data for analysis and do not unduly bias the results.

The interpretive process, performed collectively by the group, was recorded separately by each participant-researcher, and, later, as integrated by the instructor and author, involved an analysis that followed the usual steps of phenomenological inquiry (Robbins, 2006). The group read through the data with empathic engagement, broke the data into the smallest possible units of meaning, organized these meaning units into existential categories (as noted above, e.g., lived experience of body, others, time, etc.), and identified general structural themes that emerged across participants. The group utilized the eidetic technique of “imaginative variation” to help distinguish themes that were likely to be generalized by the group and to eliminate themes that were idiosyncratic (Robbins, 2006). Finally, using all of the data generating by this procedure, the author synthesized the results into a general thematic structural description of the joyful life, as it emerged through the dialogical phenomenological process. To complete this task, the author utilized the phenomenological step of translating units of meaning in the data into psychological interpretations, then synthesizing general themes identified by the students, and finally, describing the phenomenon through an integrated structural description (Giorgi, 2020).

Phenomenologists engaged in this type of research are encouraged to strive for a balance between various positions within the field which operate in tension with one other (Hopkins et al., 2017). Phenomenological descriptions are based on individual or idiographic autobiographical descriptions and interpretations. However, while the particularities of the individual descriptions have their own validity and integrity, the primary aim of phenomenological inquiry is nomothetic to the extent it aims for general insights, even as they are situated within cultural and historical horizons (Hopkins et al., 2017). Due to the small size of samples used for this approach to research, the nomothetic aim is necessarily limited, but nevertheless, eidetic analysis can provide discoveries and new insights that can be subject to hypothesis testing with quantitative strategies (Sofaer, 1999) and/or meta-analytical methods specified for qualitative research (Timulak, 2014). Despite its limitations, the bottom-up, inductive nature of phenomenology lends itself to an open-ended form of inquiry that does not rely upon pre-formulated constructs (Ihde, 2012). As such, novel insights can emerge from the data and can be generative for future research. Phenomenologists also strive to strike a balance between pure description and interpretation, and more absorbed versus more detached modes of engagement in order to grasp the meanings of first-person experiences and second-person experience data (Hopkins et al., 2017).

## Results

The results are organized in terms of the themes that emerged through the dialogical process, and involved participation by the group. Therefore, the participants’ data was interpreted together by the participants and peers who were also co-participants and co-investigators.

A dominant, shared metaphor gradually emerged from the dialog, which consisted of the image of a seed planted in the soil, breaking open, emerging from soil, sprouting and flowering as it was nurtured by the environment. This gesture was often mirrored in abstract renderings of the joyful life as a gesture expressing a motion that moved upward, outward, and expanded in its ascendence toward the top of the drawing. The metaphor also seemed to capture the tendency for participants to use earthy green and brown colors toward the bottom of the drawing, and more colorful, vibrant oranges, yellows and reds as the movement ascended toward the top of the page, against a blue or black background. Drawings deviated at times from this trope, but the image was repeated enough that it lent itself to analogies of organic growth during the group dialog.

The metaphor of a seed transforming into a seedling and bursting into life, for the participants, captured the temporal direction they understood to cut across their autobiographical, narrative descriptions. As in the redemption narrative structure identified by McAdams and McLean (2013), their stories were typically oriented from a period of crisis, tragedy or deprivation which led to the breaking down of the old self or life in order to make way for a new and emerging self that was (a) more grounded yet expansive, (b) centered yet more connected to others, (c) directed yet more open to the mysterious uncertainties of life, (d) autonomous and empowered yet uplifted and supported by others, (e) energized and hopeful for the future yet absorbed in the present moment, and (f) expansive in their desire yet grateful for what they had. These paradoxes were abundant among the discussants, and were expressed through the explicit themes, on the basis of the drawings and autobiographical narratives.

The temporal movement of the narratives seemed to be well-represented symbolically and in gesture by a seed being planted, breaking open, emerging from the soil and flowering. This analogy, for the participants in dialog, became an organizing trope that helped provide a language to bring their implicitly lived experience of joy into symbolic expression whether through image or word. It also became a shared reference among the participants which they came to more deeply understand as an image that captured an emergent, implicit structure within each of their stories and across the shared overlapping themes of their narratives.

To contextualize the general themes, the following are summary descriptions of 6 randomly chosen samples from the participants’ stories:

▪ K.M. described a personal story of being deprived of seeing her cousins who had moved to another city, and suffering a sense of loss, disconnection, and yearning in their absence. She went on to describe the day she was reunited with her cousins, and her deep appreciation for them upon their return, as they jubilantly celebrated in play together. She recalled that, during this time of play, she felt completely absorbed in the activity to the extent that she forgot about herself and had an odd sense that the present moment could last forever. She described, in turn, how she came to a renewed appreciation for her family and was grateful for their support and encouragement, which had empowered her to achieve important goals in her life. Rather than take others for granted, K.M. was prompted to consider how life is fragile and at any time we face losses in our lives that are irredeemable. This evokes for her feelings of awe, wonder, and a sense of the transcendent.
▪ J.M. surprised the group by telling a story about a sense of joy and freedom she felt the day her father died. Her father had been extremely abusive to her, and while she was removed from his care, she lived in fear that he could enter back into her life. When she heard news that her father died, J.M. felt a freedom from her past that was felt as a deep sense of relief and gladness. Her abuse by her father represented a period of her life that was characterized as broken, seemingly hopeless, and filled with terror. Upon her father’s death, she felt a sense of release from the past, could work on forgiving him, and found renewed capacities to trust and feel more connected to other people in her life. She began to look at her past challenges as essential ingredients in building the strengths that have allowed her to go on and succeed in achieving goals that really matter and to appreciate the small things in life that others might take for granted. Rather than being stuck in the past, J.M. felt safe and secure enough to be more fully present in her body, more vulnerable with others, and actively engaged and absorbed in meaningful projects. Along with these new attitudes, she felt almost a sense of unbounded freedom which uplifted and energized her, and persists as a resource she draws upon in her everyday life.
▪ K.K. described a period of longing for a romantic partner from whom she had been separated. The sense of deprivation in the absence of his presence was sometimes unbearable, and left her at times feeling shattered, alone, and detached from her surroundings as she longed only to be with him. K.K.’s transformation occurred they day they were reunited. Her experience of time was felt to be radically transformed, from the boredom of deprivation where time dragged on hour by hour, into an experience of time that was felt to be extremely quick to the extent that she was then completely absorbed and appreciative of every moment with her partner. She came to recognize a renewed capacity to take in and savor every feeling and sensation she had. This new feeling of aliveness stays with her and is cherished as a blessing.
▪ A.C. described a transformation in herself that was punctuated the day her cousin was born. The event led her to experience a sense of awe and wonder that a new life came into the world out of nothingness. At the same time, the whole event was an occasion to recognize and appreciate how close her family is, and how precious they are to her, as well as her new baby cousin. When she first held the baby, she was overcome by her beauty, studied her every move, and forgot about everybody and everything around her. She was so overjoyed, she almost cried. In her state of being awe-struck, she asked herself, “How could something be so beautiful?” She remembers every little detail of this event. She felt amazed and blessed, and had never felt this way before. She continues to cherish this moment, and it has nurtured in her the aspiration one day to be a mother and have a baby of her own.
▪ L.P. described a period of living in on-campus housing, where she and her boyfriend were very unhappy. It seemed like any mood she had was rooted in her discontent with living in the dorms. The context for her joyful transformation occurred after her and her boyfriend decided to move off campus and into a house with her sister, who was also unhappy with her living situation. When she found a perfect location for the move, she went with her boyfriend to look at the place, and experienced initial feelings of anxiousness. As they started to walk toward the place, her surroundings came vividly alive to her sensory awareness. She went on to provide detail after detail of this walk to the house, demonstrating how vividly this experience continues to live on in her memory. She felt at the time she couldn’t get enough. She had to see every inch of the area and experience all the available aesthetic quirks and beauty. Corresponding to this heightened sensory awareness, she felt a warm tightness in her chest and noticed an increased pace in their walking. She encountered future neighbors along the way, and perceived them as having a friendly demeanor that was welcoming and hospitable. That moment marked a point where her life was changed irreversibly for the better, in which her relationships with her boyfriend and her sister were blessed by their time together in the new house, and were she felt empowered to achieve goals in her life that had previously seemed out of reach. This moment of joy, which commenced a more joyful life from that point forward, lives on in her memory in great vivid detail, and has continued to serve as a source of comfort and inspiration for her, even through challenges that inevitably came along later. She remains in awe of the magical quality of that moment in time, and how it continues to nurture her even today.
▪ S.Z. described a time when he first went off to college, and suffered home sickness that was at times almost unbearable. He remembers with great joy his first return home that year, and the immediate sense of relief and well-being he felt. His anxieties were immediately suspended, and he felt comforted and protected from the world. With renewed appreciation, he greeted his dog, enjoyed hopping into his childhood bed to savor its comfortable familiarity and smell, and felt a new sense of gratitude for his family as they ate a home-cooked meal together and reconnected through conversation. This homecoming gave S.Z. a sense of belonging and a kind of warmth that he felt all over his being. It felt like being surrounded by coldness, he explained, and then suddenly entering into the warmth and welcome of an open fire. This sense of belonging allowed S.Z. to feel supported and encouraged to return to school, where he would find renewed energy and motivation to achieve success in his academic goals and to feel more connected to his peers on campus. While being at home gave him comfort, this feeling of security empowered him to take on new challenges and to succeed at school in a way that allowed him to take joy in being a fully capable human being, who is in no way hindered or handicapped. This exuberance gives him a sense that the sky is the limit and only he can decide how high he will go. He now feels confident to move on and address any new challenge.
▪ L.R. described his experience of alienation when his parents could not accept that he was gay when he came out to them at the age of 14. His parents took him to see a psychiatrist in order to force him to “change” who he is, he said. This was of course unsuccessful. Five years later, L.R. began dating his first boyfriend, and wanted desperately for his parents to meet him, accept him, and accept their relationship. So when he asked them to have dinner with his boyfriend, he was desperately hoping they would say yes. He was quite shocked to learn they actually agreed to it. At that point, though, he was still unaware of how the meeting would go, if they would approve, and just how awkward everybody might feel. As they day approached and his nerves continued, he decided there was no way he was backing out. Then, it happened – they had the dinner together. Halfway through the dinner, it occurred to him that everybody was getting along, everything was going exactly how he had hoped, and he felt nothing awkward about the situation. That was a moment of joy. He was excited about how far his parents had come in the changing of their views. He felt secure that they finally had accepted him, and on top of that, they both got along very well with his boyfriend. They most definitely approved. This ultimate approval is what brought him to joy. It changed his perceptions of who his parents are, and it allowed him to change his perception of himself. It allowed him, for the first time, to truly be comfortable in his own skin. In one sudden sweep, he felt as if the door to a bright future was open to him, expanding onto wide horizons.

The final, eidetic analysis, which identified general thematic structural features of the joyful life, was ultimately integrated and synthesized by the author into the following general themes, which aimed to stay as closely as possible to the themes that emerged within the process of the group interpretation:

▪ *Being Broken*: Initially, participants felt a sense of lacking wholeness or completion, and unworthy of happiness and joy.
▪ *Being Grounded*: Experiences of suffering and tragedy grounded the participants in a sense of humility in which they were committed with fidelity to a project that mattered to them.
▪ *Being Centered*: The participants were encouraged by others and by themselves to pursue the worthy goal, and were empowered and inspired to do so.
▪ *Breaking Open*: The participants were relieved to achieve a state which they had aspired to achieve, and all at once felt an intense, warm, powerful, radiating bursting open of the heart in the center of their being, upon this realization.
▪ *Being Uplifted*: This bursting open of the heart was felt as a light, almost weightless lifting of burdens and a sense of highness and jubilation.
▪ *Being Supertemporal*: A profound absorption in the present moment was accompanied by a feeling of limitless, unbounded space and time; a moment of profound joy was felt to persist in the background of their memory and was carried along with them indefinitely into the future.
▪ *Being Open to the Mystery*: A sense of the non-repeatability and irreversibility of the profound moment gave rise to feelings of sacredness, reverence and awe, which outlasted the emotional event and continued to permeate the background of their existence ever since.
▪ *Being Grateful*: The moment of joy was felt to be a gift of grace, or blessing, which was granted and accepted.
▪ *Opening Up and Out*: The breaking open of the self and the feeling of warmth moving up and outward from the body were felt as a boundless fecundity that called to be generously shared.
▪ *Being Together*: In the sharing of the joyful narrative, the participants experienced a sense of togetherness and community, in which a feeling of solidarity and love was given as a sense of oneness with others.

These themes were then integrated into a situated structural description of the joyful life:

Participants began their narrative descriptions in a state of brokenness, in which they felt a lack of wholeness or completion, and unworthy of happiness or joy. Participants were grounded by experiences of suffering and tragedy by which they felt a sense of humility and a calling to realize a meaningful project that was valued and viewed as intrinsically good. Participants were centered in an interpersonal or communal context through which they were encouraged – both empowered and inspired – to achieve their calling. The transformation into a joyful life began with an experience of breaking open, where they perceived that they were fulfilling an important mission or project, and this perception was accompanied by a state of emotional joy. The feeling was characterized by an intense, warm, powerful radiating bursting open of the heart in their center of their being. This feeling of joy was experienced as being uplifted, a light and almost weightless lifting of burdens and a sense of highness and jubilation. The feeling also involved a transformation in the perception of time and space, such that participants described a profound absorption in a feeling that they were unbounded by the ego-restrictions of instrumental engagement within linear, clock time and physical, geometrical space. This experience was described as having a sense of touching on a transcendent dimension beyond time and space, in which a vivid moment of joy and personal transformation would continue to pervade their experience into the indefinite future. These profound existentially grounded experiences of state joy involved a transformation of the participants into an appreciation for the mystery of being, which was punctuated by feelings of awe, reverence and gratitude, or a sense of the sacred. Participants described a feeling of gratitude for the moment of joy, which was felt to be a gift of grace or blessing that was granted and accepted. Having felt a transformation of their existence toward a transcendent dimension beyond everyday existence, the participants had the sense that their existence had undergone a profound and lasting transformation, by which the insights of their joyful moment would come to permeate the background of their experience from that moment forward. In this moment, participants felt a movement described as an opening up and out toward the transcendent, including toward other people and valued aspirations, as well as toward a transcendent, spiritual or existential dimension to existence, to which their life would become newly ordered. The breaking open of the self and the feeling of warmth moving up and outward from the body was felt as a boundless fecundity that called to be generously shared. In the sharing of joy, the participants believed they felt more deeply connected to otherness through a sense of togetherness and community. They were left with a feeling of solidarity and love that was given in their sense of oneness with others.

## Discussion

The dialogical phenomenological analysis yielded general thematic elements, and a narrative arc, that integrates aspects of hedonic, prudential, eudaimonic and chaironic variations of happiness, as identified by Wong (2011). However, in contrast to hedonic conceptions of happiness, the experience of joy did not involve the avoidance of negative emotions or tragic circumstances. On the contrary, prior to realizing the joyful life, the narratives began with accounts of being grounded in a sense of humility and unworthiness that was connected to a sense of being broken, or a lack of completion or wholeness. It was the recognition of this lack that opened the participants to “break open” with aspiration toward a valued, good end or aim. This complex relationship between joyful affect and a sense of the tragic aspect of life – with its varieties of affects from anxiety to sadness – seems to fit with prior research suggesting that states of joy tend to involve mixed emotional states including at times the complex integration of both pleasant and negative affects (Aragon, 2017).

The data in this study suggest, in addition, that a joyful life, or joyful disposition, may be initially enabled or conditioned upon a virtuous humility based on past difficulties or challenges. These challenges or difficulties may involve a strengthening of character into virtues necessary to achieve genuinely valuable or good ends. The achievement of these ends, enabled by the person’s sense of calling, has an element of active engagement of prudential well-being, but is more consistent with a eudaimonic happiness, due to its emphasis on morally or ethically valuable ends that necessitate virtue for their achievement or realization.

The chaironic sense of well-being was highly salient and clear in the qualitative data of this study. Once participants entered into a state of joy, this emotional state appeared to signal or potentiate a profound existential or spiritual transformation of the person, in which a sense of blessedness became thematic. The corresponding feelings of a sense of being gifted with a joyful life were felt as awe, reverence and/or gratitude. These qualitative insights further support prior research linking dispositional joy to more frequent experiences of gratitude (Watkins et al., 2018). The chaironic themes were also evidenced by qualities that sound almost or actually mystical in nature, such as a transformed sense of time and space, a loss of typical ego-boundedness, a sense of endless fecundity to be generously shared with others, an orientation to be grateful toward a transcendent source of the joy, and a sense of being one in communion with others beyond the self. These findings seem to validate both spiritual and existential traditions, both in the West and East, which stress that external circumstances, or ego-bound goal orientations focused on external circumstances, cannot add up to enduring joy, but only fleeting hedonic pleasure. Joy, rather, is a profound sense of grace or blessedness saturated by a sense that the experience was gifted by Being, or a being, beyond the ego or self.

Interestingly, the qualitative investigation was focused on dispositional joy understood as “the joyful life.” Yet, the narrative descriptions that were the primary basis for the data analysis, contained descriptions of both transitory state joy as well as a dispositional joy. The participants did not separate these levels of analysis, but situated state joy within a narrative structure of a joyful life. Within this narrative structure, state joy operated as an emotional state that was expressive of a moment of profound and irreversible personal transformation. These were not experiences of momentary, hedonic pleasure, nor the absence of pain. Joy, rather, operated as something like a passage from a tragic past, punctuated by a humility tempered by pain and suffering, into a transformed future in which valued goals marked the realization of a calling into a higher, more noble, or virtuous life. The capacity to achieve this higher self, and the joyful celebratory mood of its accomplishment, was seen less as the product of the person’s agency or will, but rather as a gift from beyond the self, a state of blessedness or grace.

The narrative context of the participants’ descriptions suggest a normative ordering to the levels of analysis outlined by Matthewes (2015). The momentary emotional state of joy was indeed a fleeting and powerful affective state, as described by the participants. However, this emotional state was located or situated within autobiographical stories in which this emotional state was a transitional affect, marking a type of “metanoia” – before which the person felt lost or unworthy of happiness and after which, the person came to have a sense of being blessed by a transcendent dimension to existence. After this experience, it might be said that a joyful disposition is enacted by an ongoing, background awareness of this sense of being blessed, and the motivational vitality of being directed toward a higher, moral or ethical good, operates to sustain return to similar joyful moments over the course of the lifespan. This background sense of joyfulness could perhaps be construed as something like a mood. The joyful life, as a dispositional or characterological state, encompassed the entire narrative arc, but is only realized, perhaps, with the completed transformation of the self through the momentary experience of a transformative joyful moment.

The general themes that emerged from the narrative and dialog overlap with the construct of positive orientation (Oles and Jankowski, 2018). Transformative experiences of joy carried into the future as the core of a joyful life were lived out through enhanced perceptions of the self, life and the future. The joyful life descriptions were also marked by themes found in Maslow’s (1970) descriptions of self-actualization, including more vivid perceptions of reality, feeling more accepted by oneself and others, increased spontaneity, increased autonomy, deeper appreciation, oceanic feelings, a sense of kinship with others, deeper and more profound relationships, humility, respect for others, enhanced discrimination of means and ends, and a reorientation to important values and ethics. The narratives were also ripe with themes of basic psychological needs such as enhanced autonomy, competence and relatedness to others, as developed by self-determination theory (Ryan and Deci, 2017). The narratives also yielded themes that are reminiscent of the constructs of love of life (Abdel-Khalek, 2007), resilience demonstrated through the overcoming of past challenges and adversity (Friedman and Robbins, 2012), and existential courage expressed through hardiness (Maddi, 2004). The challenge domain of hardiness is especially salient in the data, in which participants typically recalled taking on a sense of personal brokenness and deprivation, and transforming it into a positive experience. Similarly, the redemptive narrative of the bad transforming into the good was consistent throughout the narratives, and implies the joyful life for these participants had a quality characteristic of posttraumatic growth (McAdams and McLean, 2013).

Extraversion was thematic in the narratives to the extent that joyful, transformative moments tended to occur in the context of either interpersonal relationships, achievements, or both (Watson and Clark, 1997). Descriptions of profound absorption in the moment during joyful experience seemed to have some similarities to state mindfulness (Kiken et al., 2015). These moments were also characterized as moments of heightened sensory awareness, non-judgmental appreciation, and were subsequent to facing challenges in a way that could be said to be non-defensive and approach-oriented rather than avoidant. Also, the deep appreciation and sense of awe, wonder and mystery that pervades the narratives, and moments of deep gratitude and humility, reflect dispositions that overlap with hypo-egoic non-entitlement (Bankey and Leary, 2020). Spiritual well-being seemed to manifest less as overtly spiritual or religious narratives than as expressions of existential well-being, especially through personal transformation, increases in a sense of belonging and connection to others, and profound encounters with the surrounding environment, as well as through a sense of awe and mystery that may imply an encounter with the transcendent (Fisher, 1998). Finally, the observation that profound and transformative experiences of state joy became a core memory sustaining a more joyful life dovetails with evidence suggesting positive emotions facilitate and nurture various traits representative of hedonic, eudaimonic, and chaironic well-being.

### Implications for a World Challenged by the Adversity of a Pandemic

In a world besieged by a deadly virus, COVID-19, and protective measures that involve lockdowns and quarantines which can cut people off from vital social and environmental resources for coping with stress, the phenomenology of the joyful life may offer some hopeful and instructive lessons. Many people across the world are sick, fearing illness, grieving over the death of loved ones, worried about the prospects for their academic or career aspirations, struggling to make financial ends meet, being hurt in abusive relationships, feeling weighed down by addictions, suffering the torment of mental illness, and experiencing the loneliness of isolation, to name just a few challenges of our present moment in time. In other words, more people today are feeling broken – and also perhaps feeling that the world is broken, too.

The joyful life findings offer some hope. The narratives repeatedly illustrate that some of the most transformative joyful moments, which form the core of a subsequent joyful life, emerged precisely from very dark and difficult times in the lives of the participants. These transformations seemed to happen initially through gaining a sense of being grounded or planted in the soil of (often harsh) reality rather than avoiding the challenges they faced. This took some humility and a commitment (or fidelity) to an important cause or set of values. Those struggling in today’s world might consider, then, working up the existential courage to face the challenges of the moment. However, even in isolation and in a socially distanced world, the joyful life narratives suggest it is essential to find a way to connect with others. Other people help to center us, empower us, and inspire us to go on despite the darkness. Persistence in the face of potential despair may lead to a sense of breaking open, in which the person is able to feel a renewed energy and inspiration that comes with a moment of joy – a profound and transformative emotional state that is uplifting, inspires and motivates the person toward positive change. These transformations may have long-term positive consequences that will sustain the person over the long-term by providing resources to cope with future adversity and to accept challenges as opportunities for transformation and growth. Emerging from the other side of that transformation, the person might discover themselves more deeply absorbed and attentive in the present moment, more receptive to the awesome mysteries and profundity of life, more grateful and blessed for one’s life, more ready to perceive life as a gift to be shared with others, and more open to the belief that we are all in this together, potentially bounded by solidarity, love and mutuality. If these narrative descriptions of the joyful life are true to life, this personal transformation will continue as a perpetual vigil, nurturing the soul in the background of one’s awareness, as a warm feeling of coming home whenever one needs it.

### Future Directions

The qualitative analysis in this study seems to support the view that the “joyful life” construct holds promise as a central, key or guiding vision of a good life that integrates hedonic, prudential, eudaimonic and chaironic varieties of happiness to form something like a consummate expression of happiness or self-actualization. Further research should begin to develop measurements that can take these qualitative insights and transform them into reliable and valid instruments for use in future research. The findings of this study suggest that a Joyful Life Scale, for instance, would be expected to yield positive correlations with constructs such as trait gratitude, trait awe, life satisfaction, positive affectivity, psychological well-being, self-actualization, meaning in life, trait humility, spiritual well-being, and many other constructs of great interest to humanistic, existential and positive psychologists. Preliminary research in the author’s laboratory at Point Park University has begun to find evidence that may support these hypotheses (Robbins et al., 2019). Future investigations should inquire into a general personality factor that captures both the wide range of well-being represented in the qualitative data along with aspects of a health-promoting personality.

Research should also investigate whether dispositional joy may be a key to a type of resiliency that might prevent or help alleviate suffering related to trauma, or struggles with various forms of psychopathology. A joyful disposition may also be found to be linked with constructs such a mindfulness, and negatively related to constructs such as experiential avoidance. Research should also investigate whether a joyful disposition correlates with, or predicts a greater capacity to tolerate pain, or an enhanced ability to transform suffering into meaning and personal growth. Again, some preliminary evidence at the author’s laboratory suggests that a Joyful Life measure might predict physiological resiliency during a cold pressor task (a humane operational definition for physical pain) (Robbins and In ‘t Ven, 2020). Replication of these findings, and peer review of a wider and more representative sample, may continue to support these findings, at which point they can be reported. Other laboratories in the meantime, should be encouraged to begin examining similar predictions regarding the joyful disposition.

### Limitations of the Current Study

The qualitative research that forms the basis for this study has clear limitations. The use of students as voluntary participants for research, and the involvement of the researcher in the conversation, is a standard practice within the tradition of dialogal phenomenology (Halling et al., 1994). Nevertheless, such practices raise legitimate concerns about reactivity and the potential influence of the researcher on participants, even if the participants are understood to be co-researchers drawing upon their own autobiographical experience. The fact that the sharing of autobiographical details was the basis for the qualitative data, also requires humility regarding the potential for demand characteristics, such as withholding of autobiographical information in order to avoid social rejection or negative judgments from their peers or instructor. While it seems less likely that descriptions of joyful states and traits would produce the type of guilt or shame which might otherwise motivate such demand characteristics, these biases or distortions of the evidence cannot be ruled out. Also, the participants represent a homogenous sample of students in the same major at the same university within the same geographical area, and thus special caution is warranted in the generalization of these findings. The hope is that the productive generation of a phenomenological description of joy can lead to better, more valid and reliable methods for measuring joy, for the identification of its antecedents and consequences. This study is one important step in that direction.

On a final note, the qualitative findings seem to illustrate the relevance of the Joyful Life concept for a world besieged by a new and dangerous virus that has cities and towns on lockdown. Similar tragedies in the past, as already noted, have resulted in mental health consequences that cost more to quality of life than the disease itself. A hallmark of the joyful life, as a dispositional state of happiness, is that it seems to be a consummate “meaning orientation” to happiness that may signal a person’s capacity to transform tragedy and unnecessary suffering into transformative experiences. Such transformation of personal meaning seems directed toward personal growth and to stimulating prosocial motivations to give back to one’s community. If that is what the joyful life is, as suggested by our data, then the current crisis and pandemic suggests we need many more people with this disposition in the near future and for many years to come.

## Data Availability Statement

The raw data supporting the conclusions of this article will be made available by the authors, without undue reservation.

## Ethics Statement

The studies involving human participants were reviewed and approved by IRB, Point Park University. The patients/participants provided their written informed consent to participate in this study.

## Author Contributions

The sole author collected the data, integrated the results, and wrote the article reporting the findings.

## Conflict of Interest

The author declares that the research was conducted in the absence of any commercial or financial relationships that could be construed as a potential conflict of interest.
